# Developing decision model for the outsourcing of medical service delivery in the public hospitals

**DOI:** 10.1186/s12913-022-07509-1

**Published:** 2022-02-01

**Authors:** Omid Khosravizadeh, Aisa Maleki, Bahman Ahadinezhad, Saeed Shahsavari, Mohammad Amerzadeh, Nasibeh Mansouran Tazekand

**Affiliations:** 1 Social Determinants of Health Research Center, Research Institute for Prevention of Non-Communicable Diseases, Qazvin University of Medical Sciences, Qazvin, Iran; 2 Health products safety research center, Qazvin University of Medical Sciences, Qazvin, Iran; 3 Department of Epidemiology and Biostatistics, School of Public Health, Tehran University of Medical Sciences, Tehran, Iran; 4 Instructor of Biostatistics, Health Products Safety Research Center, Qazvin University of Medical Sciences, Qazvin, Iran; 5 Student Research Committee, Qazvin University of Medical Sciences, Qazvin, Iran

**Keywords:** Outsourcing, Hospital, Medical service, Decision model

## Abstract

**Background:**

The decision to outsource an activity is one of the most complex organizational decisions. This decision is also influenced by several factors and components. In order to facilitate and optimize it, for the first time in this study, a decision model for outsourcing medical service delivery in public hospitals has been developed.

**Methods:**

We conducted this cross-sectional study in 3 stages: 1) We identified the factors affecting the outsourcing decisions, 2) an expert panel identified the influential factors. After standardization, we distributed 220 questionnaires among university staff managers and heads, nursing managers, and managers of the research units, and 3) Structural Equation Model applied to evaluate the relationship between the variables on AMOS22, at 0.05 significant level.

**Results:**

Findings indicated the optimal level of all fit indices. The path coefficient between all identified factors with the outsourcing decision was positive (t > 1.96). Factors ranging from the most effective to least effective included monitoring and control, service type, human resource, economic and financial, executive capability, external environment, and terms and conditions.

**Conclusion:**

The proposed model provides unit evaluation to make the appropriate decision on outsourcing or non-outsourcing. Control and monitoring were the most determining factors. We recommend performing monitoring continuously as a guide and deterrent to error. We also recommend continuous monitoring and control over the quality of outsourced units and stakeholder satisfaction.

## Background

Stabilizing health as a fundamental principle is necessary for the sustainable development of today’s societies. As the first level of treatment referrals, hospitals must keep pace with global developments [1]. Resources are becoming more limited, and the cost structure is changing. In addition, the financial performance of public hospitals is poor, especially in developing countries [2, 3]. The answer to this problem has been sought in sources outside the organization in recent decades. In this approach, we try to use the people’s creative and persistent presence and reduce the government’s presence [4]. Outsourcing, which includes the benefits of private sector management, such as cost savings, attention to justice and social responsibility, increasing efficiency and customer satisfaction, is a solution [5, 6]. Outsourcing means contracting out some internal activities and decision-making to an external supplier. Production inputs and decision-making authority can also be outsourced [7]. The advantages of this approach include attracting outside capital, access to the global market, risk sharing, cost control, and focus on specific goals [8, 9].

### Literature review

China, India, South Africa, Thailand, Bangladesh, Turkey, and Central and Eastern European countries have benefited from outsourcing health services [2]. Laundry, Information Technology, Housing, Nutrition, Pharmacy, Laboratory, Imaging, Medical Records, Dentistry and Nursing Services, etc., have had successful outsourcing so far [10, 11]. Barati et al. Stated that outsourcing reduces costs and increases profits and satisfaction of public hospital stakeholders [12]. However, Khosravizadeh et al. Showed that outsourcing the medical records unit was not effective in reducing hospital deductions [13]. The decision to outsource an activity is one of the most complex organizational decisions. As the first part of the outsourcing process, making this decision requires identifying all the influencing factors. Besides the risks and obstacles, the benefits of outsourcing make a thorough and accurate review of this decision inevitable. Many organizations consider only the cost criterion, ignore many quality criteria, and consider failure for themselves in deciding to outsource activities. Although outsourcing has many benefits, it also has many risks that must be considered in the decision-making process [14, 15].

### Objectives

Finally, as mentioned, to achieve more benefits, many health care organizations have outsourced their activities and have considered improving the quality of their products and services. In Iran, in recent years, the government has transferred part of the services to the private sector to increase the quality of health services, increasing patient satisfaction, and reducing costs. And the results have been expressed in various studies. However, even though more than a decade has passed since implementing this approach in the health sector, there is a need for model outsourcing decisions so that its results can be used to assess the ability of hospital units to establish an outsourcing approach. However, there is still no comprehensive decision-making model in the research literature. Therefore, considering the importance of outsourcing and its role in the performance of educational and medical centers, the purpose of this study was to develop a decision model for outsourcing medical service delivery in public hospitals.

## Methods

We conducted this descriptive-analytical research using a cross-sectional plan in 2021, in the following three stages:

### Comprehensive reviewing of studies

In the first stage, we evaluated the findings of the research literature on outsourcing the educational and medical centers’ units through databases. We used data collection form tool to maintain the reliability and validity of the content, reduce bias and maintain integrity. We searched keywords “Outsourcing”, “Hospital”, “Medical service”, “Medical center”, “pattern”, “model”, “decision” and “ward” in Google Scholar, PubMed, Scopus, Web of Science, Science databases direct, Magiran, SID and Irandoc. Then, we evaluated the obtained articles qualitatively. Finally, we categorized the factors affecting outsourcing and arranged the most important ones in one division.

### Expert panel

**Fig. 1:**
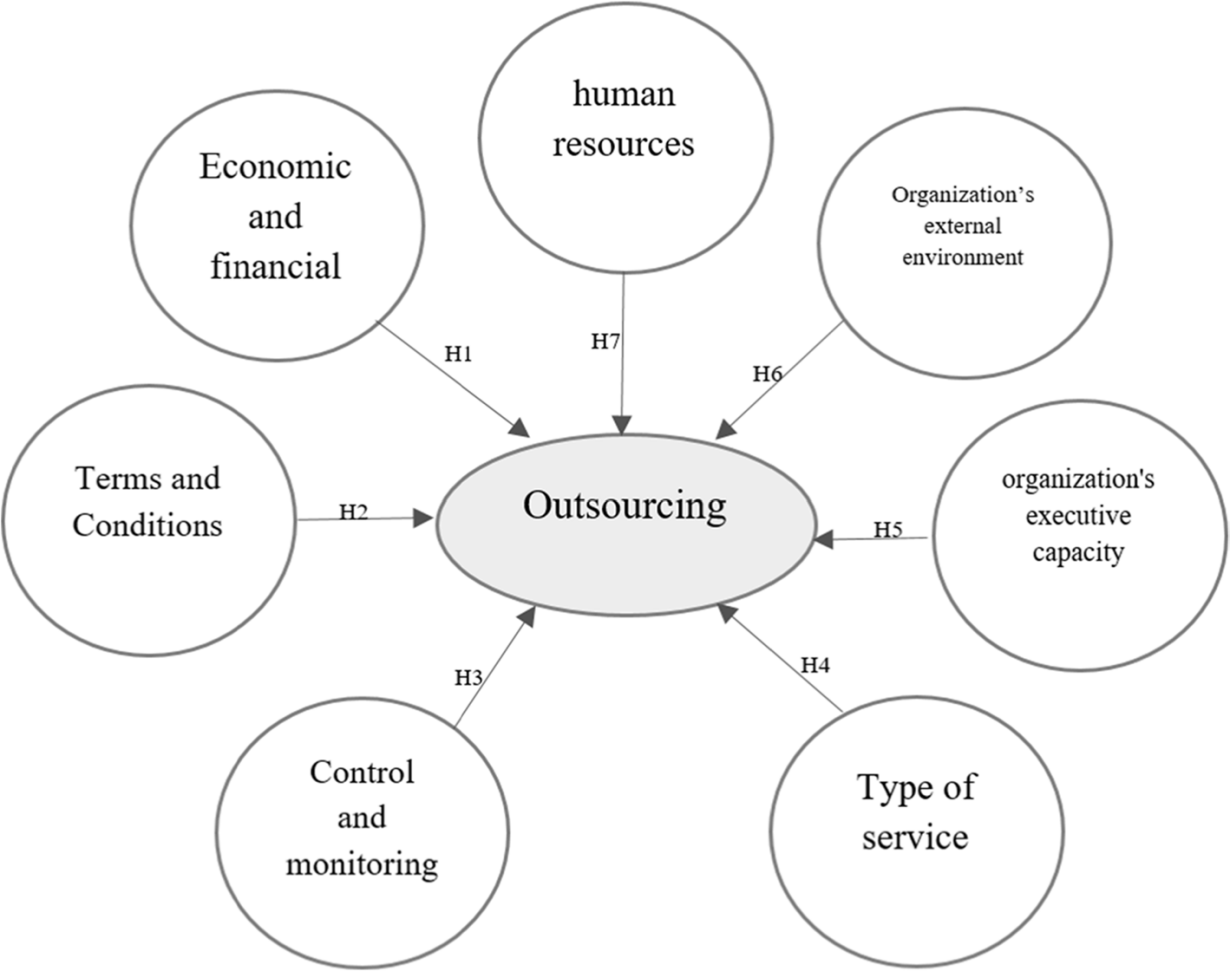
Conceptual Model of study

In the panel stage, an attempt was made to redefine the factors influencing the outsourcing of medical centers obtained from the comprehensive review stage to experts. According to this thematic background, the affective dimensions of outsourcing of Qazvin medical centers were identified by experts. The panel included people with sufficient knowledge and experience in hospital services, health management, outsourcing experience, and other related concepts. In this study, the educational and medical centers of Qazvin province were the research environment. The panel members for each of these centers also included: Head, Manager, nursing directors, Financial managers, public affairs managers, and manager of the units in which outsourcing was or is to be done. Finally, the themes and concepts of the review were compiled and categorized by experts. Figure 1 presents a conceptual model of the study. This model is the result of a comprehensive analysis of the existing research literature findings and conceptualization and classification by a panel expert panel.

Based on the literature survey [16], we proposed the following hypotheses:

- H1: The economic and financial component has a significant impact on the outsourcing decision.
- H2: The component of rules and regulations has a significant impact on the outsourcing decision.
- H3: The monitoring and control component has a significant impact on the outsourcing decision.
- H4: The service type component has a significant impact on the outsourcing decision.
- H5: The executive capability component has a significant impact on the outsourcing decision.
- H6: The external environment component has a significant impact on the outsourcing decision.
- H7: The human resources component has a significant impact on the outsourcing decision.

### Designing a questionnaire and collecting data

We developed a questionnaire using a comprehensive review and the counsellors’ and supervisors’ opinion. Expert judges assessed the face validity of the instrument. We also performed a content validity assessment by CFA.^1^ KMO^2^ index and Bartlett test were used to assess the validity of the instrument structure. We used Cronbach’s rate of 92.22 to evaluate the reliability of the questionnaire, which consisted of two parts: demographic information and outsourcing decision evaluation. The first part included five questions, including age, gender, level of education, service experience, and organizational position. In the second part of the questionnaire, we designed 44 questions in 7 dimensions. We analyzed the importance of the factors influencing the outsourcing decision with a five-point Likert scale (very low, low, medium, high, and very high).

The statistical population of this study was university staff managers and heads, nursing managers, and managers of the research units in Quds, Rajai, Velayat, Bouali, Kowsar, and 22 Bahman hospitals. We computed the sample size by multiplying the number of components by 5 (5 * 44). Therefore, we selected 220 samples. This sampling method is based on James Stevens’s logic based on selecting 5 to 15 samples per component, which is proper for multiple regression analysis applying the methods such as standard least squares, EFA, CFA, and SEM^3^ models [17]. We analyzed only completed questionnaires and excluded incomplete questionnaires. We also obtained descriptive statistics by SPSS 25.

### Validation and presentation of the final model

At this stage, we generated a final model for quantitative results. We used the SEM method to investigate the causal relationships between variables in a unified form and exhibition of the final model. This method includes five steps as follows: model representation (primary model structure), model estimation (data gathering and variable’s matrix formulation), fitness assessment (a comprehensive review of the model’s propriety, model’s feasibility, and evaluating the reform requirement), model adjustment and its interpretation. Furthermore, we utilized five indices to appraise the model fitness for SEM and CFA: We applied the χ2 index to estimate the overall model fitness and ascertain the discrepancies between appraised covariance matrices. We evaluated the approximate value of variances and covariance using the GFI^4^ through the model. A mean of the model covariance matrix to the data covariance matrix is presented with the RMSEA.^5^ We compared the model with an independent model and NFI^6^ through the CFI.^7^ We evaluated the independent model’s Chi-square values using NFI. We applied KMO test to prove the adequacy of sampling. We implemented Bartlett’s test to examine the robustness of the relationship among variables. The KMO index was 0.844 (more than 0.6), meaning the sample size was adequate for factor analysis.

Additionally, the significance level in the Bartlett test is equal to 0.001, which indicates that exploratory factor analysis is appropriate for identifying the structure. We performed these steps on SPSS 25.0 and AMOS 22 software. After all, we introduced the final validated model.

## Findings

### Participants characteristics

The average age of the participants was 39.35 years. The highest number of participants was in the age group of 31 to 40 and the lowest in over 50 years. Also, 89 of the sample were men (40.5%), and 131 were women (59.5%). Most participants had a bachelor’s degree (45.9%). Also, the level of the professional doctorate (2.7%) had the lowest frequency. Regarding the service experience of the participants, the highest number (*n* = 140, 63.6%) had 11 to 20 years of experience, and the lowest number (*n* = 38, 17.3%) had less than ten years of experience. Also, in terms of organizational positions, 90 participants (40.9%) were employed in hygiene and clinical positions (Table 1).

**Table 1:** Demographics distribution of participants

Variables	Components	Frequency	Percentage
Age	Less than 30	16	7.3
	31–40	112	50.9
	41–50	81	36.8
	More than 50	11	0.5
Sex	Female	89	40.5
	Male	131	59.5
Education level	Diploma or less	13	5.9
	Bachelor	101	45.9
	MA	82	37.3
	PhD	24	10.9
Service experience	Less than 10 years	38	17.3
	10–20 years	140	63.6
	More than 20 years	42	19.1
Organizational position	Administrative and financial	76	34.5
	Hygiene and clinical position	90	40.9
	Deputy headquarters	54	24.5

### Fit index and assessment of the model

**Fig. 2:**
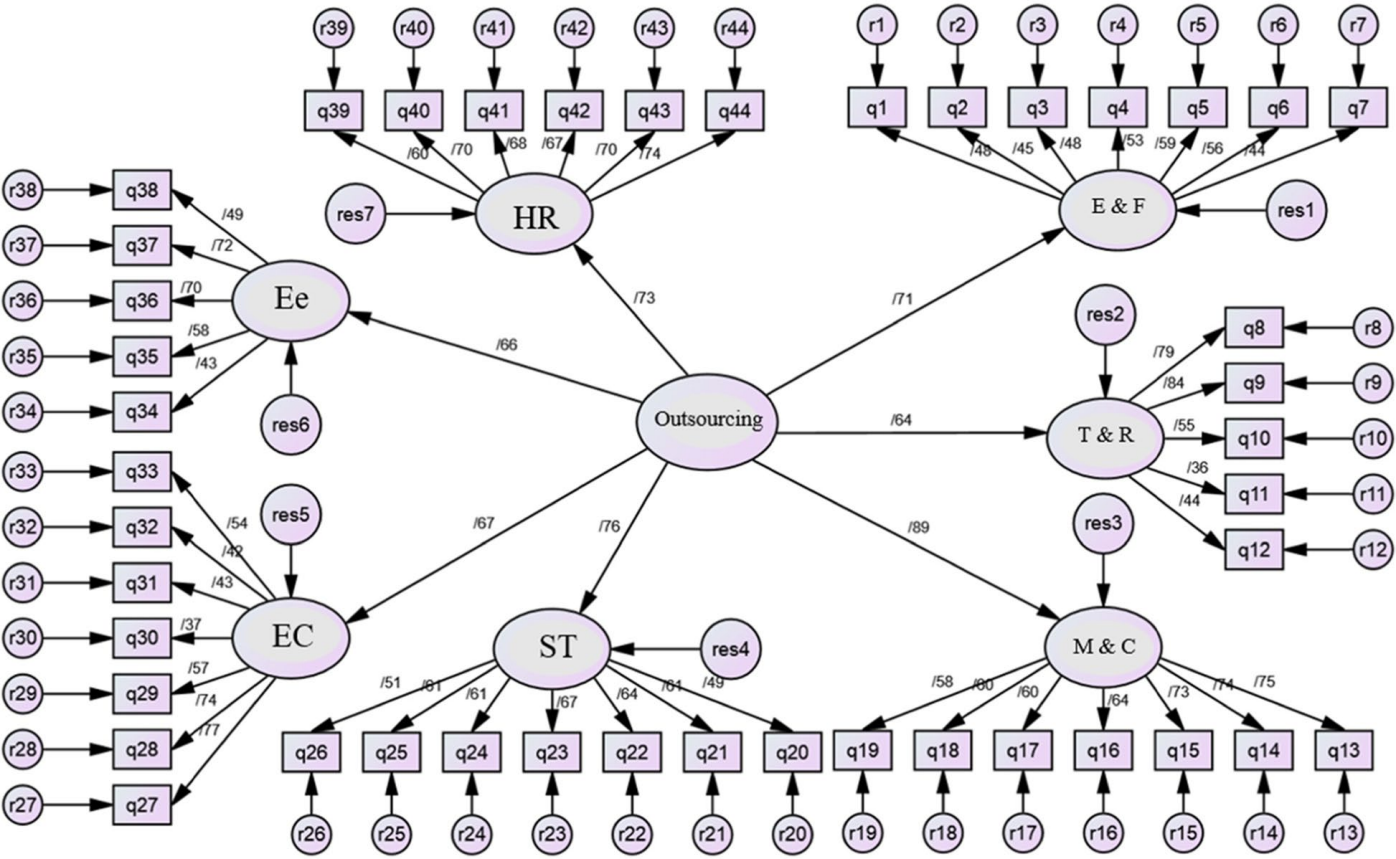
Factors for estimating the standard factor analysis of the primary model

**Fig. 3:**
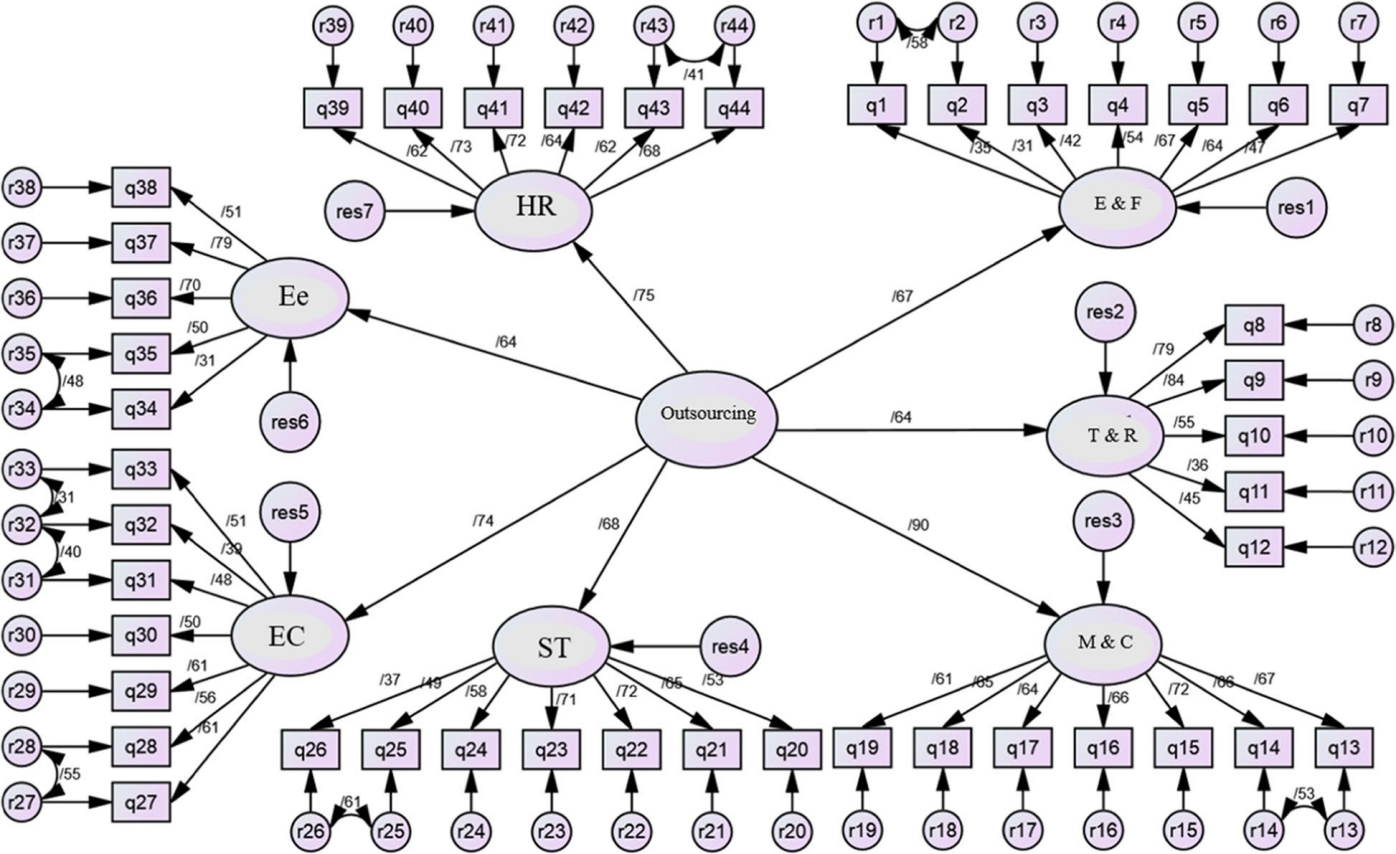
Factors for estimating the standard factor analysis of the proposed model

We applied the fitness model to examine the consistency and compatibility of the model with the extracted data. We assessed the conceptual model fitness in two stages, including the model determination segment and the structural section of the model, respectively. In the first segment, we evaluated the model for reliability and validity. We conducted a second-order factor analysis to investigate the significance of the relationship between outsourcing decisions and their factors. According to the findings of standard estimation coefficients of the second-order factor analysis of outsourcing decision, all paths were at a significant level (Fig. 2). Nevertheless, the values calculated for indices suchlike CFI, GFI, RMSEA, and chi-square to degrees of freedom, were not in the defined range showing that the achieved model did not fit enough (Table 2). Hence, we found some modifications necessary to improve the fit. We implemented these modifications in a proposed model, and the fit indices progressed (Fig. 3).

**Table 2:** Comparison of fitness indices in the primary model and the proposed model

proposed model	Limit	Index
2.376	Less than 3	χ2/df
0.904	Higher than .90	GFI
0.79	Less than .08	RMSEA
0.911	Higher than .90	CFI
0.956	Higher than .90	NFI

### Statistical hypothesis testing

Structural equation modelling analysis revealed a significant correlation between outsourcing decisions and their related factors. We used the value of T-Student statistics corresponding to each coefficient to measure the significance of the estimated paths, reported in Table 3. To confirm the hypothesis at the 95% confidence level, the value of the t-test corresponding to that test must be higher than 1.96. Considering that the value of t-statistic for all components of the path coefficients is more significant than 1.96, all path coefficients of the hypothesis are significant. Therefore, all hypotheses of this research have been confirmed.

**Table 3:** Results of structural equation modeling of Dimension in final model

symbols	Hypotheses			Mean	SD	t statistic	Path coefficient	*P*_value_	Results
H1	**Economic and financial**	**--->**	**Outsourcing**	26.359	3.582	4.614	0.709	***	Accepted
H2	**Terms and Conditions**	**--->**	**Outsourcing**	18.100	3.100	4.550	0.643	***	Accepted
H3	**Monitoring and control**	**--->**	**Outsourcing**	26.904	4.271	4.752	0.889	***	Accepted
H4	**Type of service**	**--->**	**Outsourcing**	26.750	3.747	4.101	0.758	***	Accepted
H5	**Executive capability**	**--->**	**Outsourcing**	26.718	3.508	4.508	0.669	***	Accepted
H6	**The external environment**	**--->**	**Outsourcing**	19.150	2.674	3.545	0.664	***	Accepted
H7	**Human resources**	**--->**	**Outsourcing**	23.481	3.422	4.296	0.732	***	Accepted

## Discussion

This study aims to provide a model for evaluating the outsourcing of educational and medical centers in Qazvin province. For this purpose, we conducted a comprehensive review. We examined the extracted components according to hospital experts to identify and conceptualize the final model. Finally, all experts agreed upon seven dimensions (economic and financial, legal, control and monitoring, service type, the executive capability of the organization, the external environment of the organization and human resources) in the form of 44 components. Then we developed, evaluated and approved quantitative research tools. Finally, we built and modified the model to fit correctly.

Economic and financial factors with a path coefficient of 0.709 directly affect about 70% of outsourcing decision changes. Many studies confirm this finding [14, 16, 18, 19]. This includes freeing up resources to invest in other activities, comparing contractors’ offers, costs during contract execution (inflation, unforeseen costs), service costs, projected financial and budgetary resources, cost-benefit analysis and the projected benefits of services. The most common reason for hospitals to outsource is to reduce costs. Therefore, analyzing the external supplier in terms of economics, service costs, financial feasibility of the contract is important for the organization and prepares health service providers to be more competitive. Meanwhile, the production of services by a contractor who provides only a specific service, occurs more efficiently. Scale savings also occur under these conditions.

On the other hand, according to Dehghanipodeh et al., outsourcing frees up internal resources to spend on more strategic matters. Also, the ultimate goal of outsourcing is to increase profitability, flexibility and reduce the investment risks of the organization. An outsourcing decision is made when the organization is confident that outsourcing will improve its financial status and productivity [20].

Terms and conditions factors with a path coefficient of 0.643 directly affect about 64% of outsourcing decision changes. Rahman et al. and Raeissi et al. have emphasized the importance of this dimension [3, 14]. This factor includes the transparency and flexibility of the provisions of the contract, the legal validity of the contractor, the laws and regulations of the Ministry of Health and the laws governing the contractor. A contract that sets out conditions such as time, cost, rewards, and risks for both parties creates a secure relationship. In addition to transparency, such a contract should include evaluation indicators and control methods [21]. The contract is best managed and monitored when it pays attention to the details of service levels and metrics based on cost reduction, service delivery, service improvement, and user satisfaction. As a result, it is essential to consider the inclusion of oversight rules in any outsourcing contract.

Monitoring and control factors with a path coefficient of 0.889 directly affect about 88% of outsourcing decision changes. Many studies have pointed out this dimension [16, 22, 23]. This includes monitoring and supervising the strict implementation of contract provisions, focusing on key points of monitoring and evaluating contractors ‘performance, having a valid database for recording contractors’ performance information, defining a structured standard for monitoring and evaluating contractors ‘performance, and managers’ mastery of skills for evaluating the performance of contractors, the ability to measure the continuous improvement of service quality and the ability to measure the satisfaction of other units and the client of the outsourcing target unit’s performance. Service providers must apply their control in both tangible and intangible ways. This control is more severe in the early stages but should generally be continuous. In the further steps, it continues as relationship management. This leads to the correct implementation of the contract and consequently reduces costs (such as evaluation and negotiations costs) and achieves a competitive advantage. In this regard, defining standards, monitoring key points, access to valid data, continuous quality assessment, and satisfaction are necessary [3, 22].

Type of service factors with a path coefficient of 0.758 directly affects about 75% of outsourcing decision changes. Many studies have pointed out this dimension [16, 23–25]. This includes the tangibility of the service, the existence of service delivery protocol, the degree of service connection with the client, the degree of interdependence of services, the strategic importance of services, the complexity of processes and the need for service in the mission of the organization. The nature of services such as complexity, structural integrity, required resource capacity, availability of equipment and tools, controllability, and popularity in the organization culture are determining factors [16, 24, 25]. We must first identify the organization’s goals, key capabilities, and main missions to determine the activities that can be outsourced. Any non-core activity can be outsourced. The activities that are the primary mission of the organization are not usually outsourced. Some services inside and outside the organization can better provide efficiency, quality, cost-effectiveness, added value and customer respect.

Executive capability factors with a path coefficient of 0.669 directly affect about 66% of outsourcing decision changes. This includes the support of financial and operational managers, the participation and support of influential people, attention to internal capabilities and key capacities, agility and flexibility of the organization against change, intra-organizational coordination and the extent of conflict of interest. Many studies have pointed to this dimension, including Kavosi et al. [16]. In today’s rapidly changing environment, organizations need to have the necessary flexibility to maintain a competitive position. Because doing all of an organization’s processes internally requires resources, expertise, and attention, often not sufficiently available, outsourcing provides this flexibility. Executive capability to outsource requires the support of managers, participation of all stakeholders, cooperation and coordination of units, and conflict resolution [1, 26–28].

The external environment factors with a path coefficient of 0.664 directly affect about 66% of outsourcing decision changes. Many studies have pointed to this dimension [16, 23, 25].. This includes the political, economic and cultural status, the existence of reputable and qualified contractors, the extent to which competitive capacity is achieved, the existence of modern technology, the type of technology and the facilities used by the contractor. Achieving maximum potential benefits and minimum risk requires selecting a qualified supplier [1, 19]: the more suppliers, the less risky the choice. Maintaining a competitive position through outsourcing [16, 19, 23] responds to the rapid growth of technology, the explosion of knowledge, the growing demand of customers, the changing appearance of diseases, the increasing pressures of scarce financial resources, and the achievement of productivity. Equipping the contractor with the latest equipment, facilities, resources, and technology in the world is also necessary [3, 22, 24].

Human resource factors with a path coefficient of 0.732 directly affect about 73% of outsourcing decision changes. This includes training and empowerment of employees, measuring the knowledge and skills of employees, the status of qualified personnel within the organization, coordination of employees and the degree of release of human resources for internal works. Many studies have pointed to this dimension [1, 16, 19]. Whereas processes performed by specialized personnel are more efficient and less risky, outsourcing will take place if the staff’s expertise and skills and the possibility and qualification of training are low. On the other hand, when outsourcing is communicated to employees, there is naturally anxiety, worry, a sense of job insecurity, and consequently a decrease in motivation and performance. In such a situation, creating an outsourcing organizational culture leads to the acceptance of outsourcing, and employees seek their own and the organization’s interests in implementing outsourcing. This culture, along with providing transparent information to employees, strengthens the coordination of internal staff with outsourced staff [26, 27].

The most influential factor in outsourcing health services was monitoring and control (88%). Monitoring and control ensure the fulfilment of obligations at the level of setting standards. Errors in evaluating other factors such as economic and financial factors, selection of the appropriate contractor, type of outsourced service, etc., occur as deviations and problems in the outsourcing process. Therefore, continuous monitoring and control is a factor that allows early detection of errors and even their correction.

Limitations of this study include 1) We made the research tool with the opinions of experts that may lead to mental attitudes and memory biases, 2) Lack of access to some databases in the comprehensive overview section.

## Conclusion

Outsourcing is increasingly being used as a simple and economical executive tool. The proposed model allows quantitative and qualitative evaluation of units to maintain or outsource. Despite the many benefits of outsourcing in terms of cost reduction and efficiency improvement, sometimes it may be ineffective or even harmful. Therefore, if outsourcing is not possible, internal upgrades are recommended. Numerous factors, from economic and financial to service and contractor, affect the success of outsourcing. Control and monitoring and the type of service are the most determining factors. Therefore, we recommend that outsourcing decisions, including the choice of outsourced service and the contractor, be taken from an expert group. In addition, monitoring should be done continuously as a guide and deterrent to error. Continuous monitoring and control over the quality of outsourced units and stakeholder satisfaction are also essential. In addition, the whole outsourcing process should be done within the framework of national and organizational laws and regulations. Also, to survive a successful quality outsourcing, it is recommended that the provision of services based on specific procedures, specialized training and empowerment of employees, identification of qualified staff, and attention to employee motivation be on the agenda of the managers of the units. Finally, to maximize executive capability, senior executives should coordinate units and manage conflicts effectively.

## Data Availability

All data generated or analyzed during this study are included in this article.

## Abbreviations

E & F: Economic and financial
T & C: Terms and Conditions
M & C: Monitoring and control
ST: Type of service
EC: Executive capability
Ee: External environment
HR: Human resources

## Footnotes

1 confirmatory factor analysis

2 Kaiser Meyer Olkin

3 Structural equation modeling

4 Goodness of Fitness Index

5 Root Mean Square Error of Approximation

6 Normed Fit Index

7 Comparative Fit Index

## References

[1] Ali Nejad H (2021). The outsourcing model in the Ministry of Health and Medical Education with fuzzy Delphi technique in order to improve the quality of health services with emphasis on hospitals. Management Strategies in Health System.

[2] Barati O (2017). A study of the status before and after outsourced pharmacies of Shiraz University of Medical Sciences in 2014: a short report. J Rafsanjan Univ Med Sci.

[3] Raeissi P, Sokhanvar M, Kakemam E (2018). Outsourcing in Iranian hospitals: findings from a qualitative study. Int J Health Plann Manag.

[4] Franco M, Rodrigues M, Silva R (2021). The viability of outsourcing in Organisational performance: Benefits Risks.

[5] Mujasi PN, Nkosi ZZ. A comparative analysis of the costs and benefits of outsourcing. Insourcing cleaning Services in a Rural Hospital in Uganda. Open Pharmacoecon Health Econ J. 2018;6(1):9–20.

[6] Mujasi PN, Nkosi ZZ (2019). Exploring perceptions, motivations, and practices regarding outsourcing support services by general hospitals in Uganda: a mixed methods study. Int J Health Plann Manag.

[7] Meng W, Xuejiang W (2019). Research on logistics outsourcing decision-making model based on cost and competence. in Proceedings of The First International Symposium on Management and Social Sciences (ISMSS 2019).

[8] Arastoozadeh F, Torabipour A (2017). Comparison of services' quality in outsourced and non-outsourced clinical laboratory in Ahvaz University hospitals, 2016. J Healthc Manag.

[9] Yazdanpanah B, MOMENI MH (2017). Studying the levels of training outsourcing from the perspective of training experts in governmental departments of the southern Khorasan.

[10] Borowska M (2020). Selected factors determining outsourcing of basic operations in healthcare entities in Poland. Health Policy.

[11] Sarabi Asiabar A (2021). Economic consequences of outsourcing in public hospitals in Iran: a systematic review. J Health Adm.

[12] Barati O (2019). Outsourcing in Shiraz University of Medical Sciences; a before and after study. J Egypt Public Health Assoc.

[13] Khosravizadeh O (2019). Do medical records outsourcing affect insurance deductions? An interrupted time series in Qazvin’s trauma center. J Surg Trauma.

[14] Rahman HU (2021). Empirical investigation of influencing factors regarding offshore outsourcing decision of application maintenance. IEEE Access.

[15] Kaveh Pishghadam H, Esmaeeli H (2021). A system dynamics model for evaluating the firms’ capabilities in maintenance outsourcing and analyzing the profitability of outsourcing. Scientia Iranica.

[16] Kavosi Z (2018). Factors influencing decision making for healthcare services outsourcing: a review and Delphi study. Med J Islam Repub Iran.

[17] Stevens JP (2015). Structural equation modeling. Applied multivariate statistics for the social sciences.

[18] Rowshan M (2020). Identifying and prioritizing effective factors on outsourcing in public hospitals using fuzzy BWM. Hosp Top.

[19] Shirdeli M (2018). Presenting a model to evaluate factors affecting outsourcing of health information technology services. Acta Inform Med.

[20] Poudeh HD (2019). Determining and prioritizing the factors influencing the outsourcing of complex product systems R&D projects employing ANP and grey-DEMATEL method (case study: aviation industries organization, Iran). Technol Soc.

[21] Joudaki H, Heidari M, Geraili B (2015). Outsourcing of hospitals services: lessons learned from the experience. J Health Based Res.

[22] Mousazadeh Y (2013). Identifying and prioritizing Hospital’s units for outsourcing based on related indicators: a qualitative study. J Health.

[23] Hanafizadeh P, Zareravasan A (2020). A systematic literature review on IT outsourcing decision and future research directions. J Glob Inf Manag.

[24] Suweero K, Moungnoi W. Outsourcing decision factors of building operation and maintenance services in hospital business. Eng Appl Sci Res. 2016;43:439–42.

[25] Perera KH, Jayaratne P (2017). Derivation of a decision model on outsourcing in Sri Lanka using analytic hierarchy process.

[26] Navidi A, Taghipour ZA, Ahmadi SAA (2017). Presenting the educational and research activities outsourcing model in organizations governmental (case study: great tehran electrical distribution company).

[27] Farhoodi H, Abdi B, Aghamohammadi V (2016). Critical success factors identification and prioritization of information systems in National Iranian oil Products Distribution Company: a strategic planning approach. Farayandno.

[28] Martínez‐Sánchez Á, José Vela‐Jiménez M, de Luis‐Carnicer P, Pérez‐Pérez M. Managerial perceptions of workplace flexibility and firm performance. Int J Oper Prod Manage. 2007;27(7):714–34.

